# Supplementary material for: Systematic comparison of high-throughput single-cell RNA-seq methods for immune cell profiling
Source: BMC Genomics. 2021 Jan 20;22:66. doi: 10.1186/s12864-020-07358-4 (PMC7818754; doi:10.1186/s12864-020-07358-4)
Supplement: Supplementary file 1 — Additional file 1: Supplement Figs. 1–7. [file 12864_2020_7358_MOESM1_ESM.pdf]

Supplementary Information

Additional file 1:

Supplement Figure 1 Read-depth normalization and cell classification: (a) Determined read distribution peaks used for read-depth normalization. (b) Normalized read distribution per experiment for classified cells. (c) Normalized read distributions per experiment and cell type. (d) Correlation of gene counts to bulk-RNA-seq data for all experiments. Top cell identifiers by numbers of reads (2x number of expected cells) were included in the plot.

Supplement Figure 2 Cell metrics of pre-normalized data**:** (a) Transposed log-log empirical cumulative density plot of the total counts of each CID. The curve is shifted in each plot to highlight each cell and cell type that was determined by correlation to bulk RNA-seq using correlation thresholds determined in downsampled data. Knee and inflection points are indicated. (b-d) Boxplots show number of reads, number of UMIs, and number of detected genes per cell without normalization of library read depth. (e) Cell type composition of each sample.

Supplement Figure 3 Library efficiencies: (a) Estimates of multiplet rates for each experiment based on number of CIDs with significant numbers of transcripts from human and mouse. Rates were adjusted to account for variable recovery of human and mouse cells. (b) Fraction of mapped bases aligning to intergenic (red), intronic (green), coding (purple), and untranslated (UTR) (cyan) regions by sample.

Supplement Figure 4 Transcript detection sensitivity: (a) Distributions of numbers of unique molecular identifiers (UMIs) and genes across all classified cells, or by cell-type. Read distribution was most consistent for EL4 cells across samples. (b) Models for dropout rate by expression level and cell type. A left shifted curve indicates higher sensitivity. (c) Dropout rates for mouse genes by expression level in bulk RNA-seq for EL4 cells. Solid lines indicate modeling curves for all methods. (d) GD_{50}, the FPKM at which the dropout rate is expected to be 0.5, for dropout models by cell type. A low GD_{50} indicates high sensitivity.

Supplement Figure 5 Correlation of single-cell RNA-seq to bulk RNA-seq. Number of detected genes were plotted as a function of the highest correlation coefficient *r* of CIDs above the inflection point to bulk RNA-seq.

Supplement Figure 6 Differentially expressed (DE) genes. (a) Number of significant DE genes using the Mann-Whitney-Wilcoxon test or a negative binomial generalized linear model between EL4 and IVA12 cells. Error bars represent the 95% confidence interval from ten random sub-samplings of cells from each method. The total number of significant DE genes are plotted in red, the number of DE genes with > 1.5-fold difference in expression in bulk RNA-seq (5,868 genes) are plotted in cyan. (b) Number of significant DE genes (FDR < 10^{-4}) between all EL4 and IVA12 recovered cells for each method. Genes with > 1.5-fold change in bulk RNA-seq data are plotted in cyan with the remainder in red. (c) Comparison of mouse gene fold changes in single-cell RNA-seq vs bulk RNA-seq for contrasting EL4 and IVA12 cells. Genes are colored by highest expression value (FPKM) in bulk RNA-seq data. Black lines indicate an absolute fold change of 1.5. The numbers of genes with an absolute fold change greater than 1.5 in both single-cell and bulk data for each quadrant are indicated in blue. While highly expressed genes in green correlate well between single-cell and bulk data, lowly expressed genes in red show little difference in expression in single-cell data. (d) Median gene expression (FPKM) in bulk sequencing for all significant DE genes (red) or DE genes with > 1.5-fold difference in expression in bulk RNA-seq (cyan) from ten random sub-samplings of cells are shown. Error bars represent 95% confidence interval. (e) Distribution of gene expression level (FPKM) in bulk RNA-seq for DE genes identified using all cells for each method. All significant DE genes are plotted in red and DE genes with > 1.5-fold change

in bulk RNA-seq data are plotted in cyan. Distribution of expression levels for all genes with > 1.5-fold change in bulk data are plotted in gray.

Supplement Figure 7 Peripheral blood mononuclear cell (PBMC) single-cell analysis**.** (a) Distribution of cells projected onto an annotated PBMC CITE-Seq reference dataset by method (10x 3' v3 – red, 10x 5' v1 - cyan) (b) Number and fraction of cells by cell classification for each method. (c) Heatmap of scaled expression across cells plotted for differentially expressed genes (expressed in at least half of cells, ln fold change > 0.25). Cell classes with more than 100 cells were randomly subsampled to 100 cells. (d) Distribution of number of reads for each cell class and method.

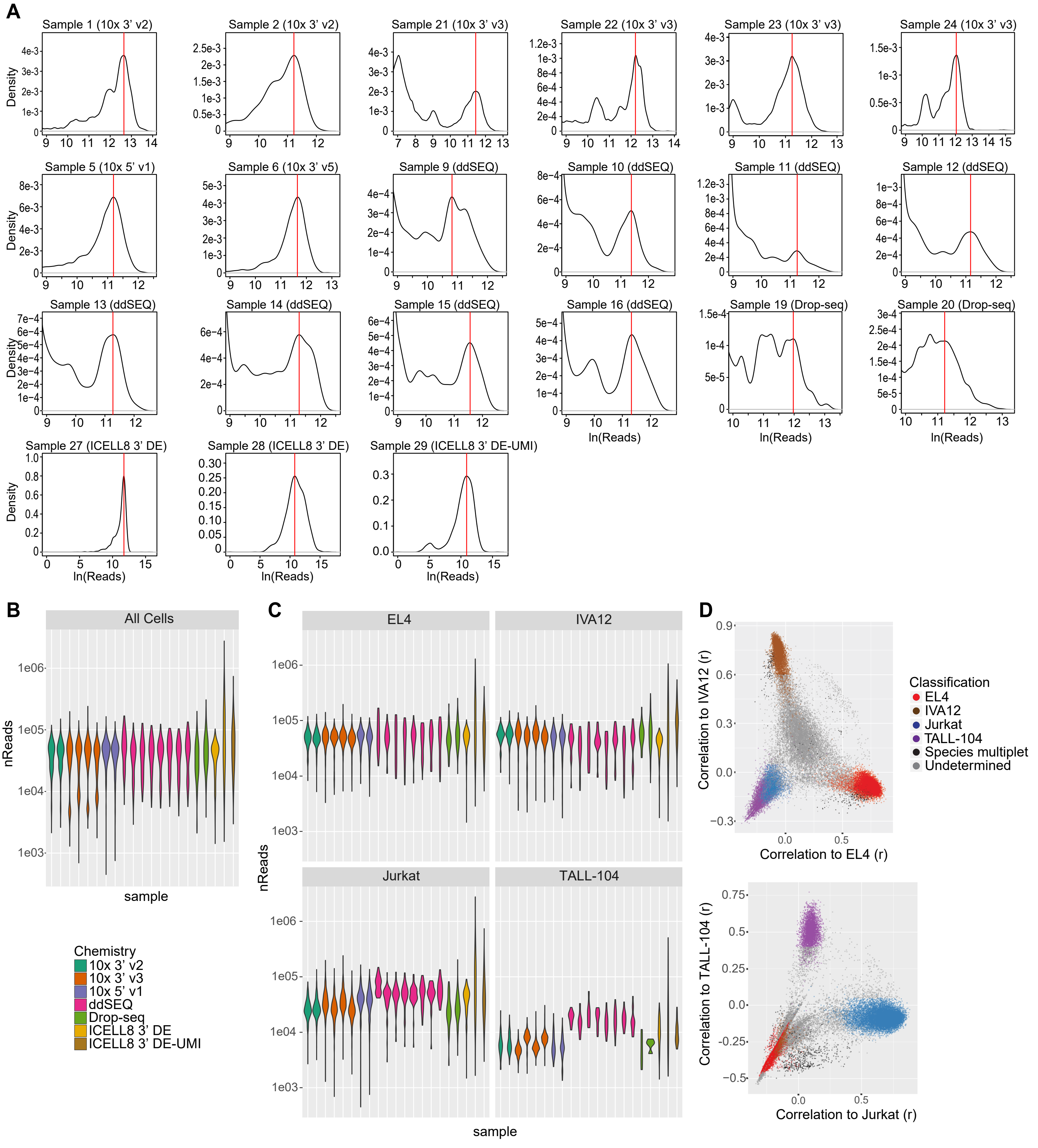

Supplemental Figure 1

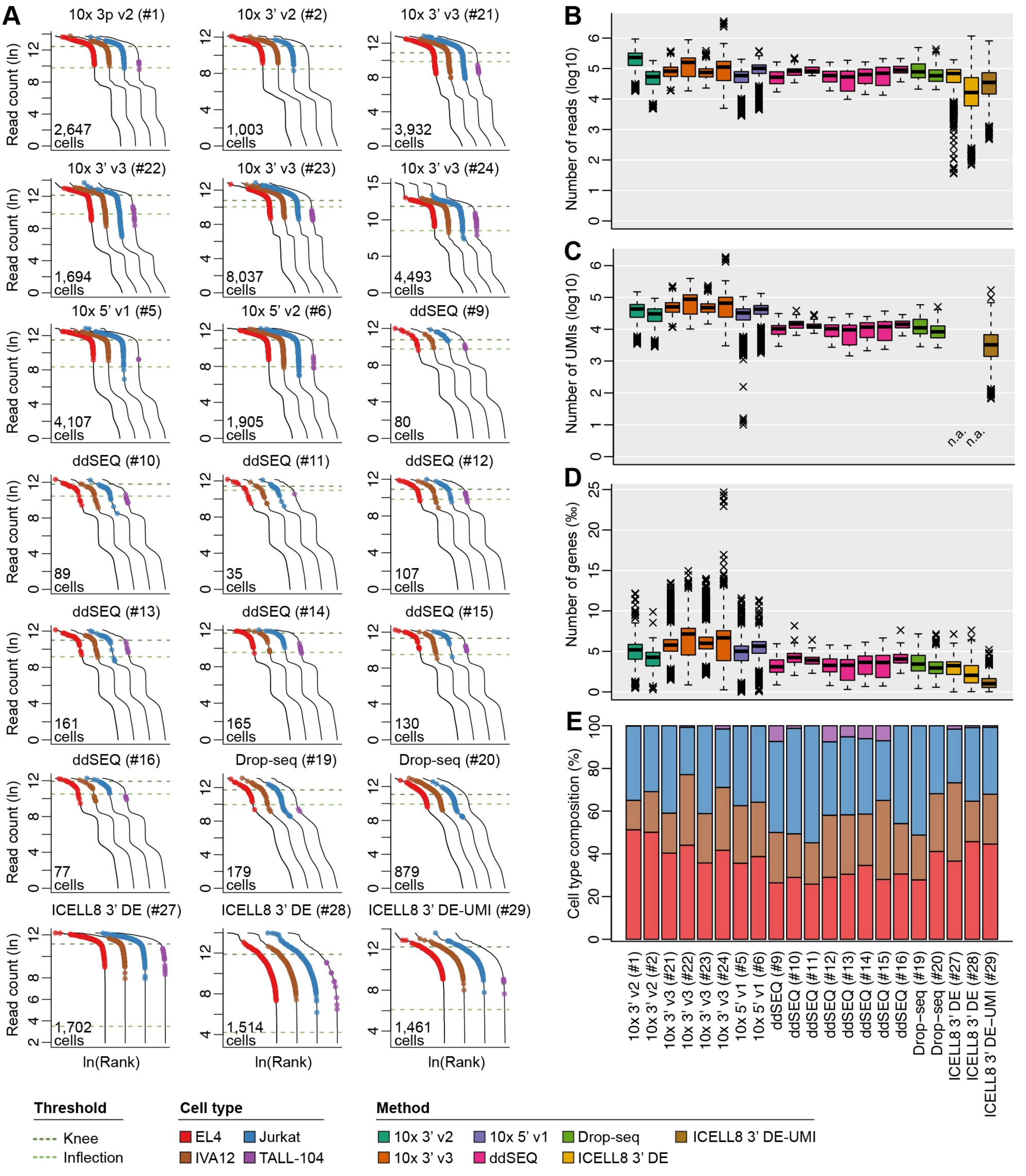

Supplemental Figure 2

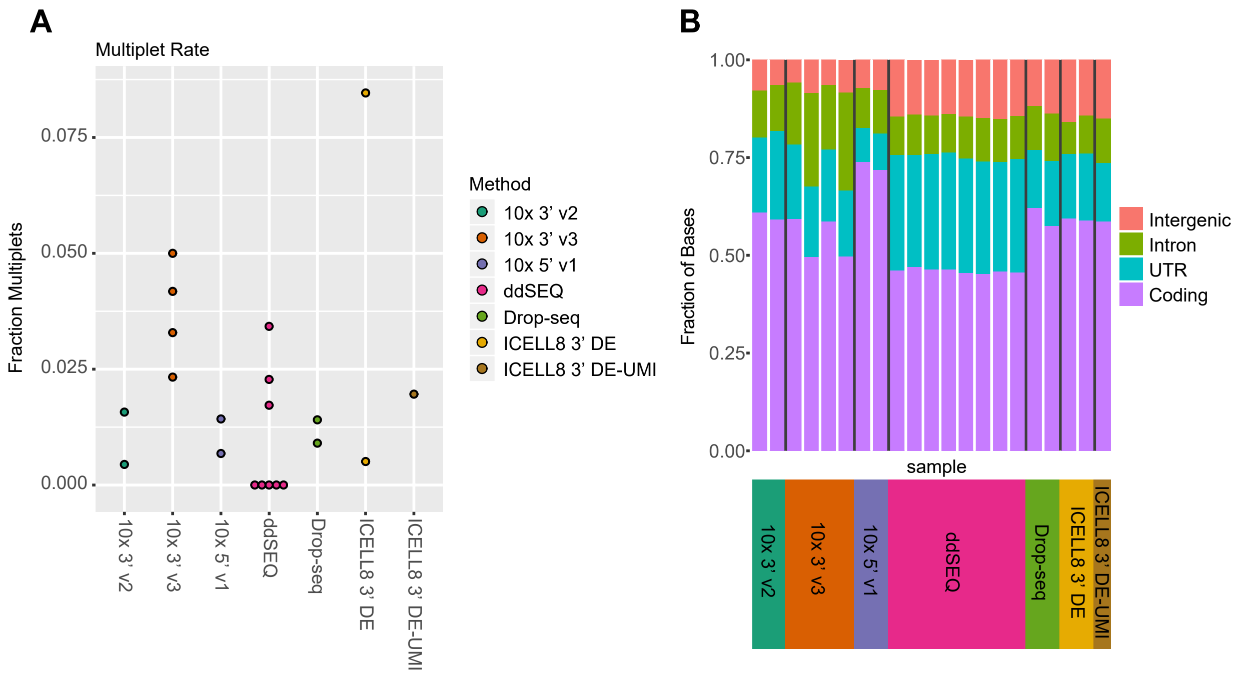

Supplemental Figure 3

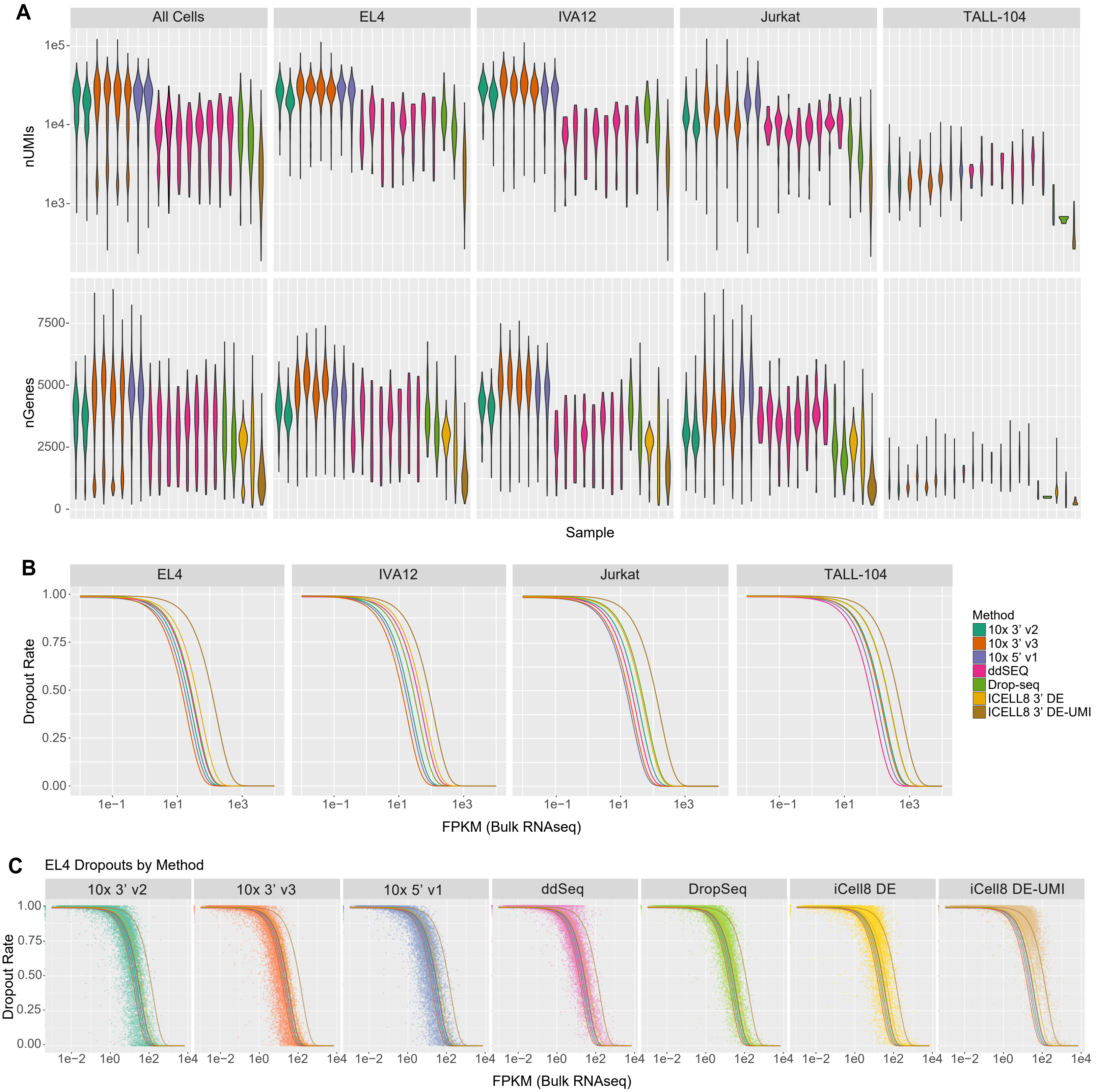

Gene Detection 50 (GD_{50})

D

	10x 3' v2	10x 3' v3	10x 5' v1	ddSEQ	Drop-seq	ICELL8 3' DE	ICELL8 3' DE-UMI
EL4	20.18	13.64	16.79	25.04	26.75	37.88	112.15
IVA12	18.67	12.26	15.77	36.84	27.47	45.79	82.16
Jurkat	28.51	16.92	14.80	21.73	43.73	39.98	114.09
TALL-104	119.49	110.10	85.21	61.44	205.16	199.72	411.22

Supplemental Figure 4

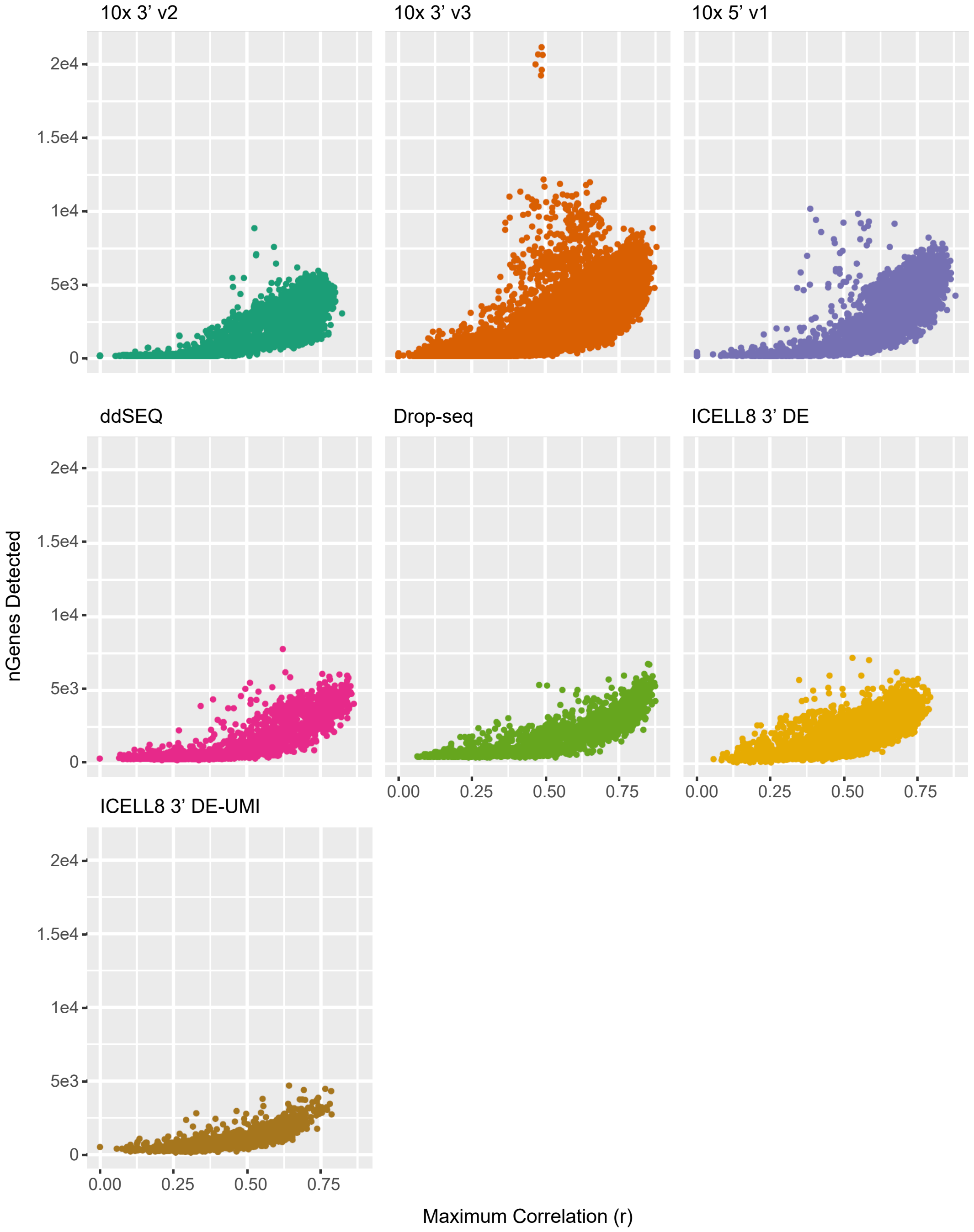

Supplemental Figure 5

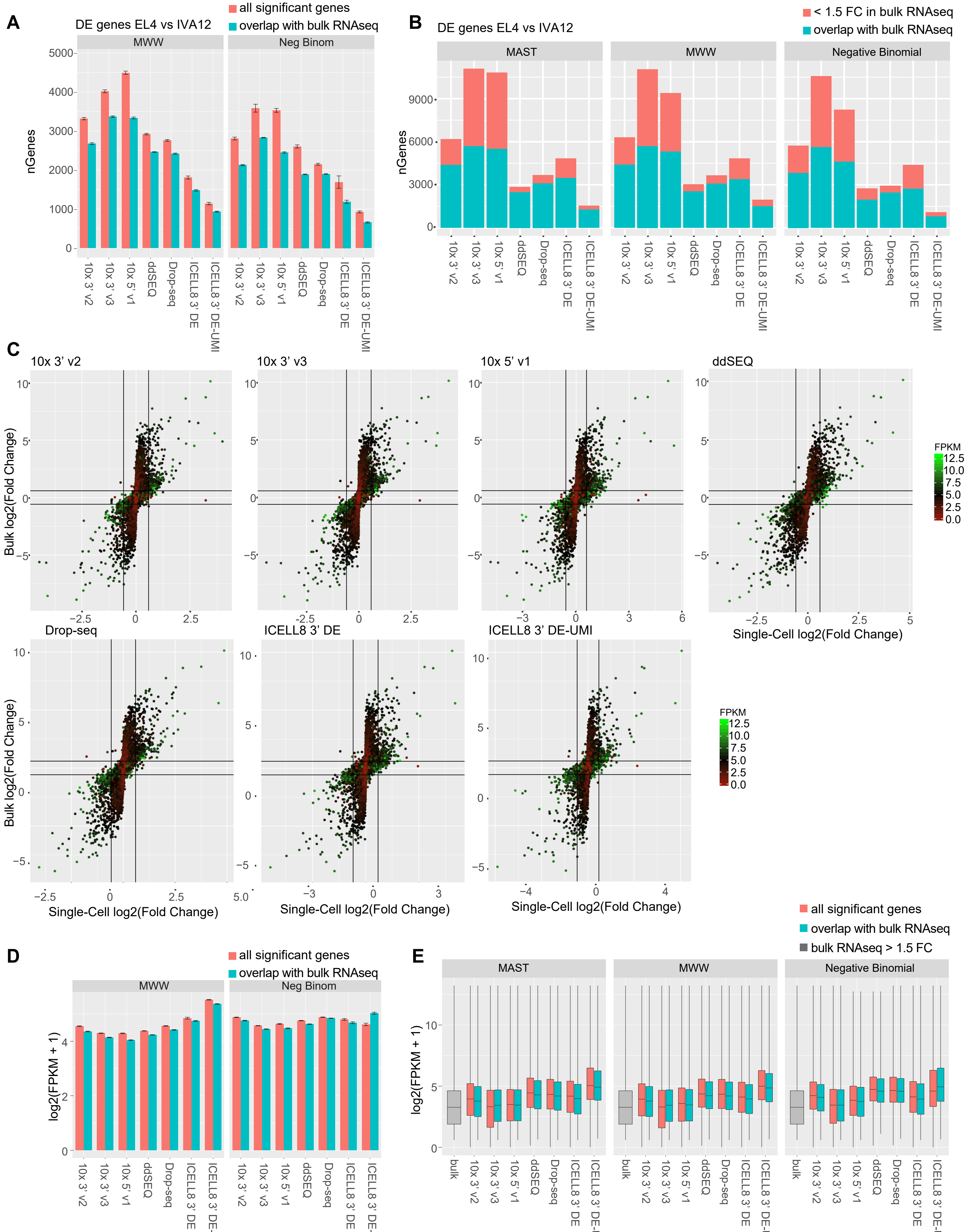

Supplemental Figure 6

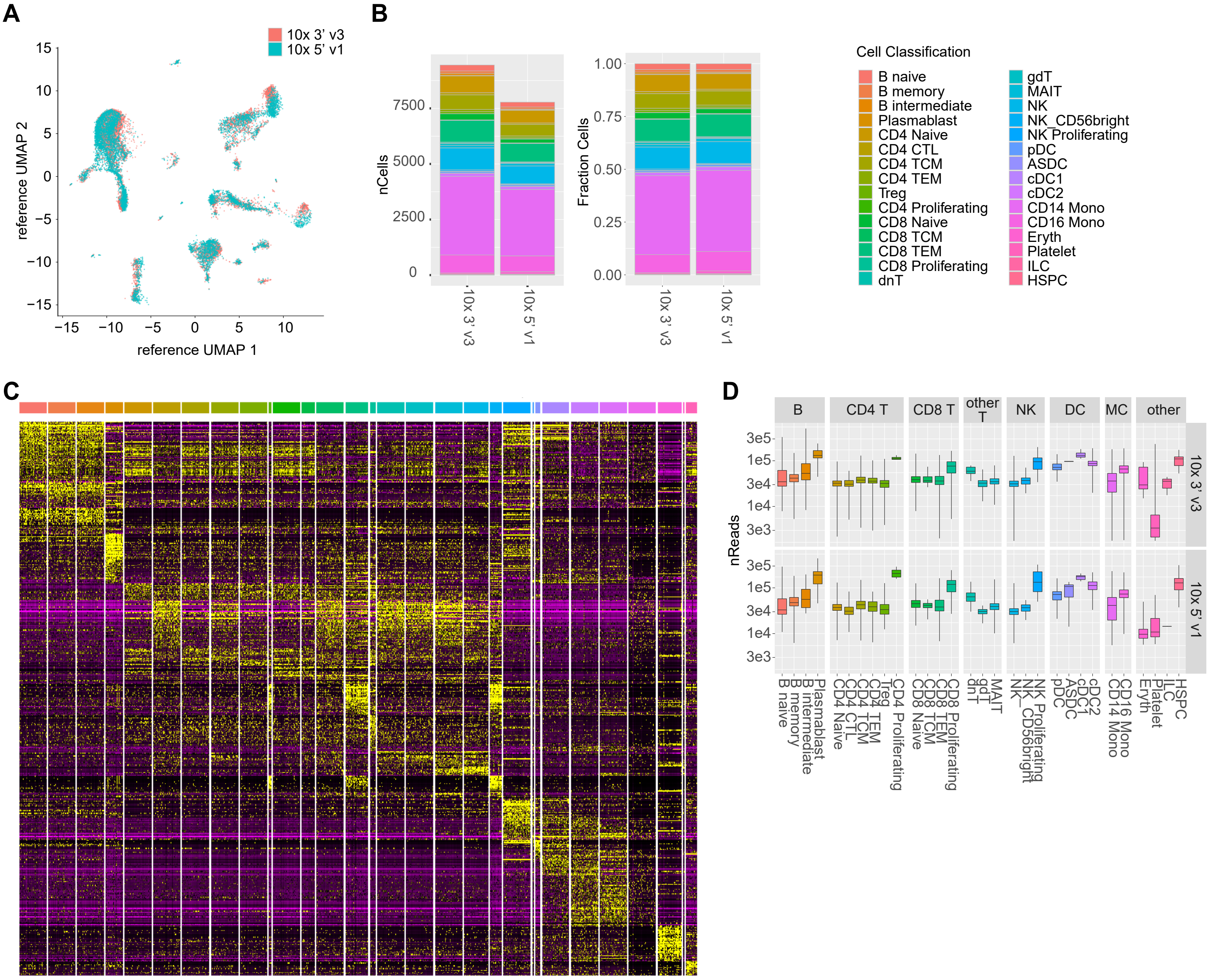

Supplemental Figure 7
